# Relationship between Subtalar Joint Stiffness and Relaxed Calcaneal Stance Position in Cerebral Palsy Children with Valgus Deformities

**DOI:** 10.1155/2018/6576108

**Published:** 2018-04-30

**Authors:** Wei Chen, Jie Yao, Yang Yang, Xiaoyu Liu, Lizhen Wang, Fang Pu, Yubo Fan

**Affiliations:** ^1^Key Laboratory for Biomechanics and Mechanobiology of Ministry of Education, School of Biological Science and Medical Engineering, Beijing Advanced Innovation Centre for Biomedical Engineering, Beihang University, Beijing, China; ^2^National Research Center for Rehabilitation Technical Aids, Beijing, China

## Abstract

Relaxed calcaneal stance position (RCSP) is an important index in the correctional treatment of foot valgus deformities for cerebral palsy (CP) children. However, patients with similar RCSP showed diverse outcomes when accepting similar treatment, as the corrective resistance of subtalar joint (STJ) could be quite different. This study aimed to investigate the relationship between STJ stiffness and RCSP in different loading conditions. 38 valgus feet of 19 CP subjects were included in the study. A reposition force was applied beneath the STJ and pushed the foot from pronated position to neutral position. The STJ stiffness was calculated as the slope of the line fitting the force-displacement data. Correlations between the STJ stiffness, RCSP, and composite spasticity index (CSI) were analyzed. The spearman correlation coefficient indicated that STJ stiffness had no correlation with RCSPs, yet it had negative correlation with the change of RCSP under difference loading conditions (ΔRCSP_1w−0w_ and ΔRCSP_0.5w−0w_). STJ stiffness was also correlated with the composite spasticity index (CSI), implying that this index had an advantage in reflecting the mechanism of valgus deformity and should be considered as a necessary measurement of foot valgus in CP children. The present method for quantification of STJ stiffness could improve the accuracy in the diagnosis and classification of foot deformity and may help increase the understanding of the biomechanical factors in foot deformity rehabilitation.

## 1. Introduction

Planovalgus is the most common foot deformity in cerebral palsy (CP) children [1–4]. It is characterized as hindfoot valgus during weight bearing, associated with a pronated midfoot and flattened longitudinal arch [5–7]. Long-term valgus deformity would lead to the navicular drop [8]. The head of the talus will become the main bearing areas and cause pain during walking [1, 9, 10]. The cause of valgus deformity is multifactorial, including soft tissue imbalance, muscle spasticity, and joint malalignment [10–12]. Therefore treatment method should be based on the inherent mechanism of the valgus deformity. Inappropriate treatment may lead to unsatisfactory outcome and complications [6, 13, 14]. A precise assessment of the valgus severity usually plays an important role in the treatment options.

Relaxed calcaneal stance position (RCSP), radiographs, hindfoot valgus angle, and valgus index are commonly used methods to assess the foot valgus severity [5, 15–17]. These assessment indexes are based on the foot anatomical morphology under the static condition and have significant correlation with each other [16, 18]. In particular, RCSP, defined as the angle between the heel bisection and a vertical line, is technically convenient and has been widely used in ankle deformity orthopedics [19, 20]. However, there is still dissent regarding the efficiency of these morphological indexes, and the treatments based on these indexes also resulted in unsatisfactory outcomes [21].

Subtalar joint (STJ) stiffness is a biomechanical index that reflects the corrective resistance of the foot planovalgus. Clinicians have found that patients with similar morphological valgus could have very different corrective resistance [22–24]. Treatment programs without considering the corrective resistance may fail to achieve adequate outcome. However, there is still a lack of quantitative definition of the corrective resistance. In manual reposition therapy, the corrective resistance is conventionally evaluated by experience. Interobserver reliability is low when inexperienced observers carry out the tests [25]. Our previous study has developed a method to quantify the apparent stiffness of subtalar joint (STJ), which could indicate the corrective resistance of the foot valgus deformity in CP children [26]. However, the relationship between the STJ stiffness and morphological valgus severity in CP children remains unclear. The insights gained through the relationship may lead a way to a better understanding of the mechanisms of the foot deformity and an optimization of the rehabilitation therapy.

This study aimed to investigate the relationship between the STJ stiffness and the morphological characteristics of valgus foot (RCSP) in CP children. Since there was no exact definition of STJ stiffness, the STJ stiffness was defined as the resistance in response to a reposition force, which was widely used for valgus deformity correction in CP children. To compare the STJ stiffness with RCSP from the perspective of foot deformity pathology, the correlations between the composite spasticity index (CSI) and the valgus parameters (STJ stiffness and RCSP) were also investigated.

## 2. Materials and Methods

### 2.1. Subjects

A total of 19 diplegic CP children (10 males and 9 females, age: 6.3 ± 2.21, height: 1.20 ± 0.12 m, and weight: 22.90 ± 6.20 kg) participated in this study. The inclusion criteria were as follows: (a) the spasticity was lower than level III according to the modified Ashworth scale [27]; (b) valgus deformity occurred at the hindfoot; (c) there was no surgery history of the foot or ankle; and (d) the heel could be manually flattened at the time of assessment. Informed written consent, in accordance with clinical protocols approved by Beihang University, was obtained from the parents of each subject.

### 2.2. STJ Stiffness Measurement

**Figure 1:**
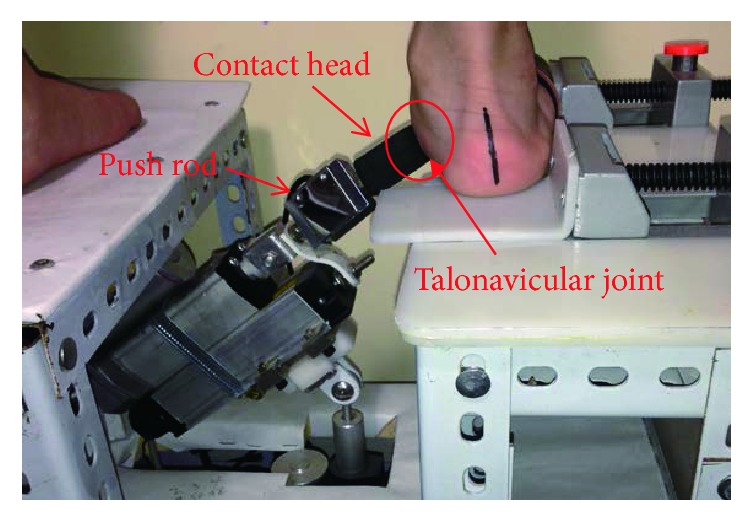
Overview of the device to measure the STJ stiffness.

**Figure 2:**
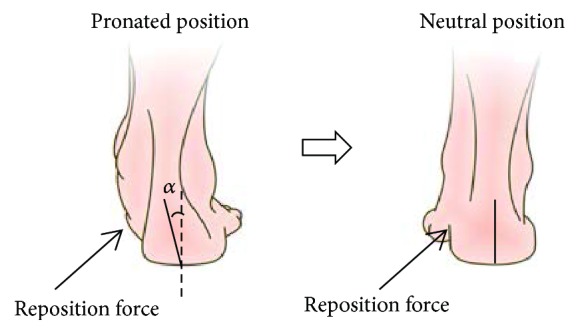
Schematic view of the measurement.

The STJ stiffness was measured with the device designed in our previous study (Figure 1) [26]. To standardize the position of the measurement, the subject was required to stand naturally on the platform; the ankle flexion angle was maintained at 90°. The foot was kept flat, and the lateral sides of foot were kept in contact with the fixed board. As shown in Figure 2, a reposition force was applied beneath the medial site of the talus with a horizontal angle of 40 degrees, which is a widely used reposition force for valgus deformity in CP children. The initial position of contact head was placed just in contact with the skin at the talus. Then the contact head pushed the STJ with a constant speed (6 mm/s) to restore the foot from pronated position to neutral alignment. To make the reposition process more comfortable, the contact head of the pushing rod was designed as a semicircular ethylene-vinyl acetate copolymer part. The force sensor (accuracy in 0.01 kg, range in 200 N, JLBS-v, System engineering Technology Co., Ltd., China) and the displacement sensor (sampling frequency in 12 Hz, KTC-50 mm, Boshi Technology Co., Ltd., China) were fixed at the rod to record the force and displacement. Data were recorded in real time and transferred through a USB connection with a computer. Each foot was measured three times with 20-minute intervals. The force-displacement data was recorded.

### 2.3. RCSP Measurement

The subject was positioned in relaxed standing. The RCSP was defined as the angle between the calcaneal bisection line and the line perpendicular to the ground [19] (*α* in Figure 2). Since RCSP will be influenced by the loading condition of the lower limbs, three conditions (weight-bearing, half-weight-bearing, and non-weight-bearing) were considered in the experiment. In weight-bearing condition, the subject stood with one foot, keeping the other foot backward off the ground, and the subject's hand could touch a handrail gently to balance himself; in half-weight-bearing condition, the subject stood in a relaxed bipedal stance with their feet apart as wide as an adult's fist; in non-weight-bearing condition, the subject sat with the feet fist-width apart, RCSP was measured on the unsupported feet. For each condition, the foot was measured three times with the protractor.

### 2.4. CSI Grading

Spasticity is a critical cause of valgus deformity, and CSI is a commonly used clinic index to assess the spasticity degree in CP children [28, 29]. To compare the STJ stiffness and RCSP from the perspective of foot deformity pathology, CSI of each lower extremity was measured. The CSI subindexes (consist of Achilles tendon jerk, gastrocnemius tone, and ankle joint clonus) were graded, respectively.

### 2.5. Statistical Analysis

Spearman correlation matrices of STJ stiffness, RCSPs, and CSI were calculated by correlation analysis with the SPSS software (IMB, US). A correlation coefficient different from 0 and a significant level (*p* value) < 0.05 indicate a considerable correlation. A two-way random average measure intraclass correlation coefficient (ICC(2, *k*)) was used to assess the repeatability of the methodology [30]. A value greater than 0.75 indicates the desirable repeatability.

## 3. Results

### 3.1. CSI

The CSIs of 19 children with 38 feet were listed in Table 1. CSI is comprised of tendon jerk, muscle tone, and clonus. The mean score of tendon jerk was 2.4 ± 0.59. The mean score of muscle tone was 4.6 ± 1.90. The mean score of clonus was 1.2 ± 0.36. The mean CSI was 8.2 ± 2.65.

### 3.2. STJ Stiffness

**Figure 3:**
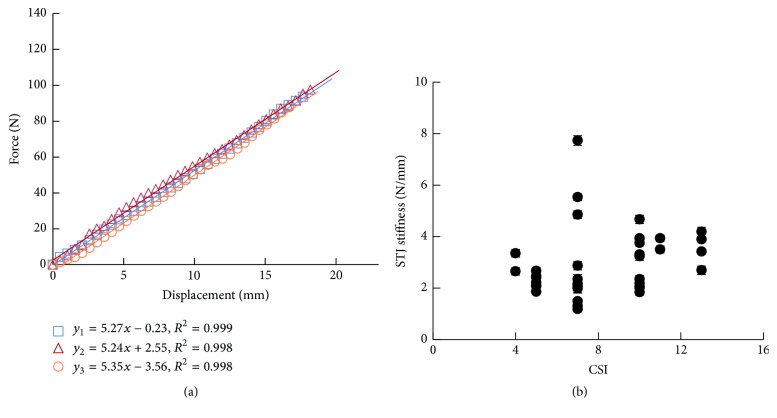
STJ stiffness of 38 feet. (a) Force-displacement data of reposition process in three measurements of one subject. A line was used to fit the force-displacement data with least squares fitting algorithm. The STJ stiffness was defined as the slope of the line. (b) STJ stiffness-CSI data of 38 feet.

The force-displacement data of the reposition process in one foot was shown in Figure 3(a). A least-square-fit line was calculated according to the force-displacement data. The STJ stiffness was defined as the slope of the line. The stiffness was 5.27, 5.24, and 5.35 N/mm, respectively, in three measurements of the foot. The accuracy of the fitting (*R*^2^) was 0.999, 0.998, and 0.998 N^2^, respectively. The STJ stiffness-CSI data was shown in Figure 3(b). The STJ stiffness was scattered within the range of 1.18 N/mm to 7.73 N/mm. The mean stiffness of 38 feet was 2.94 ± 1.27 (mean ± SD).

### 3.3. RCSP

**Figure 4:**
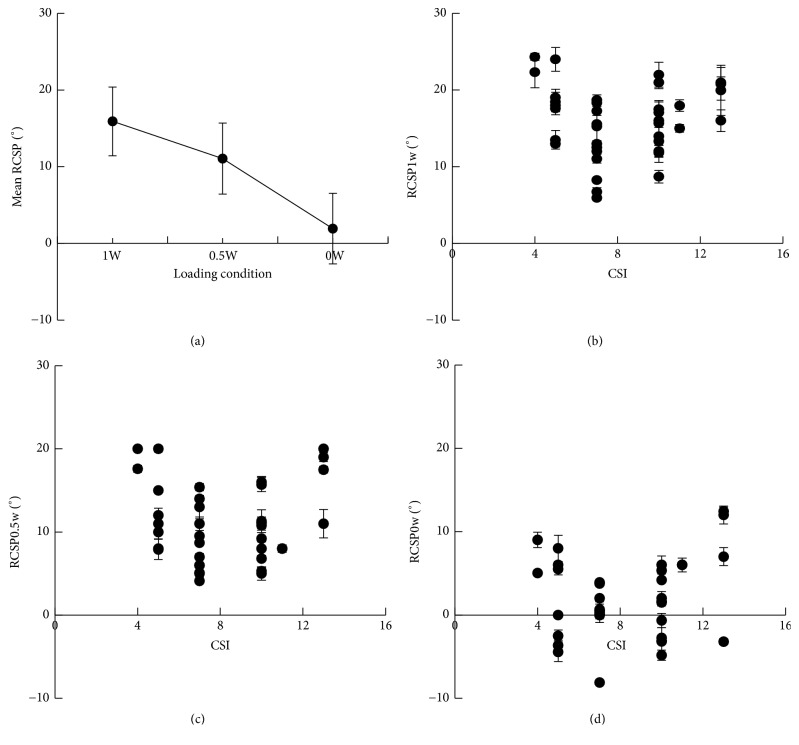
RCSP of 38 feet. (a) The mean RCSP decreased with the body weight loading. (b) The RCSP_1w_-CSI data. (c) The RCSP_0.5w_-CSI data. (d) The RCSP_0w_-CSI data.

**Figure 5:**
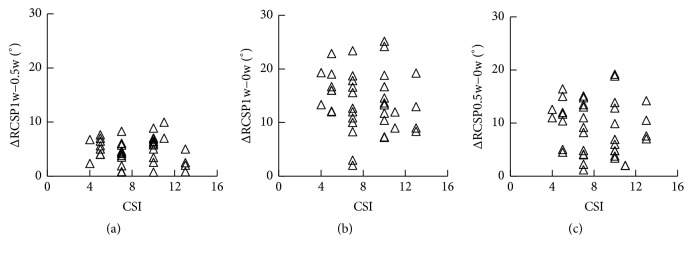
Influence of loading condition on RCSP. (a) ΔRCSP_1w−0.5w_ = RCSP_1w_ − RCSP_0.5w_. (b) ΔRCSP_1w−0w_ = RCSP_1w_ − RCSP_0w_. (c) ΔRCSP_0.5w−0w_ = RCSP_0.5w_ − RCSP_0w_.

The RCSP decreased with the body weight loading (Figure 4(a)). The mean RCSP under weight-bearing condition (mean RCSP_1w_) was 15.91° ± 4.49°, under half-weight-bearing condition (mean RCSP_0.5w_) it was 11.06° ± 4.64°, and under non-weight-bearing condition (mean RCSP_0w_) it was 1.92° ± 4.61°. The RCSP-CSI data of 38 feet under three loading conditions were shown in Figures 4(b)–4(d). To further investigate the influence of loading alteration on the RCSP, the differences between RCSP_1w_ and RCSP_0.5w_ (ΔRCSP_1w−0.5w_), between RCSP_1w_ and RCSP_0w_ (ΔRCSP_1w−0w_), and between RCSP_0.5w_ and RCSP_0w_ (ΔRCSP_0.5w−0w_) were calculated (Figure 5).

**Figure 6:**
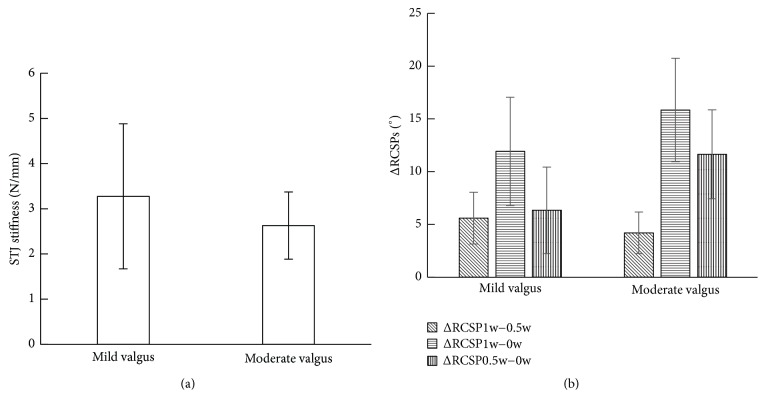
STJ stiffness and ΔRCSPs in mild and moderate valgus groups. (a) Mean STJ stiffness. (b) Mean ΔRCSP_1w−0.5w_, ΔRCSP_1w−0w_, and ΔRCSP_0.5w−0w_.

The feet were further divided into two groups. 0° < RCSP_0.5w_ ≤ 10° was classified as mild valgus group (group 1), and 10° < RCSP_0.5w_ ≤ 20° was classified as moderate valgus group (group 2) [31]. The mean STJ stiffness and ΔRCSPs were shown in Figure 6. With RCSP_0.5w_ increasing, mean STJ decreased and ΔRCSP_1w−0.5w_ decreased, while ΔRCSP_1w−0w_ and ΔRCSP_0.5w−0w_ increased.

### 3.4. Correlation Analysis

**Figure 7:**
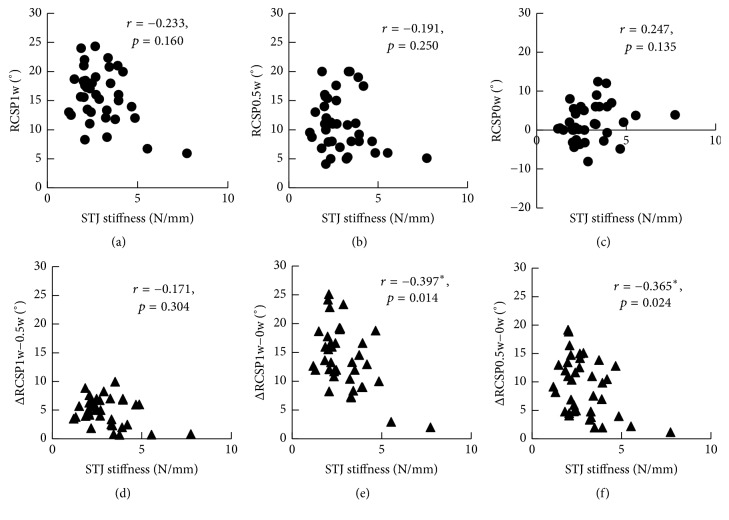
Correlations between STJ stiffness, RCSPs, and ΔRCSPs. (a) Correlation between STJ stiffness and RCSP_1w_ (*r* = −0.233; *p* = 0.160). (b) Correlation between STJ stiffness and RCSP_0.5w_ (*r* = −0.191; *p* = 0.250). (c) Correlation between STJ stiffness and RCSP_0w_ (*r* = 0.247; *p* = 0.135). (d) Correlation between STJ stiffness and ΔRCSP_1w−0.5w_ (*r* = −0.171; *p* = 0.304). (e) Correlation between STJ stiffness and ΔRCSP_1w−0w_ (*r* = −0.397^*∗*^; *p* = 0.014). (f) Correlation between STJ stiffness and ΔRCSP_0.5w−0w_ (*r* = −0.365^*∗*^; *p* = 0.024).

The correlations between STJ stiffness, RCSPs, and ΔRCSPs were shown in Figure 7. STJ stiffness has no correlation with RCSPs and ΔRCSP_1w−0.5w_ but has negative correlations with ΔRCSP_1w−0w_ and ΔRCSP_0.5w−0w_.

A Spearman correlation matrix of all 8 variables was shown in Table 2. STJ stiffness was positively correlated with CSI (*r* = 0.343^*∗*^; *p* = 0.035). RCSP_1w_, RCSP_0.5w_, and RCSP_0w_ were correlated with ΔRCSP_1w−0w_ and ΔRCSP_0.5w−0w_. ΔRCSP_1w−0.5w_ was not correlated with any other variables. Furthermore, the correlations between CSI subindexes (tendon jerk, muscle tone, and clonus) and the valgus indexes were shown in Table 3. The STJ stiffness was correlated with muscle tone (*r* = 0.335^*∗*^; *p* = 0.040) and clonus (*r* = 0.392^*∗*^; *p* = 0.015). Tendon jerk was not correlated with any other variables. ICC(2, *k*) was 0.995 and the lower and upper bounds of the 95% confidence interval were 0.991 and 0.997, which indicate desirable repeatability of the methodology.

## 4. Discussion

This study investigated the relationship between indexes of the foot valgus in spastic CP children, the STJ stiffness, and RCSPs. The character of STJ to resist deformation was contributed by talocalcaneal joint, talonavicular joint, and the related connective tissues. The STJ stiffness plays an important role in foot kinetics, especially during weight-bearing activities. However, there was no exact definition of STJ stiffness previously. In the present study, the STJ stiffness was defined as the stiffness in response to an external force with a horizontal angle of 40 degrees, which was a widely used reposition force for valgus deformity in CP children. In the present study, CSI had a significant correlation with STJ stiffness (*r* = 0.343; *p* = 0.035), whereas it had no correlation with RCSPs (RCSP_1w_, RCSP_0.5w_, and RCSP_0w_). Since CSI is a reliable measure of lower extremity spasticity, which is one of the critical factors of foot valgus in CP children, the findings implied that STJ stiffness could be more directly related to the gait and foot function than RCSP.

Besides spasticity, STJ stiffness is also related to the abnormities in joint structure and tissue mechanical property. When the foot is at the weight-bearing position, a pathological STJ could lead to valgus deformity symptoms including the pronated position of the calcaneus, medial bulging of the navicular tuberosity, abduction of the forefoot, and a reduction in the height of the medial arch [32]. The degree of the STJ abnormities has a direct relationship with the severity of the foot deformity. Furthermore, the reposition ability of STJ under the external force was also considered as a reference for the development of treatment in clinics. However, this reposition ability was usually estimated with qualitative manual testing based on the therapists' experience [22, 25, 33]. The estimation accuracy is erratic and thus may lead to an unsatisfying treatment. The present study presented a quantitative method for the characterization of STJ stiffness, which could provide the basis for the development of treatment program.

RCSP was chosen as the morphological parameter to indicate the degree of foot deformity in this study, because the morphology-based indexes (e.g., RCSP, radiographs, valgus angle, and valgus index) have high correlations between each other [16, 18], and RCSP is more convenient to measure in clinics and has no risk of radiation. Compared to the STJ stiffness, RCSP is statically determined from the perspective of morphology. However, there is still dissent regarding the efficiency of morphological indexes; the treatments based on morphological indexes also resulted in unsatisfactory outcomes [21]. The present study divided the feet into mild and moderate valgus groups according to the degree of RCSP; the results showed that the mean STJ stiffness in mild valgus group was even greater than that in moderate valgus group. The present study further demonstrated that the RCSPs (RCSP_1w_, RCSP_0.5w_, and RCSP_0w_) had no significant relationship with STJ stiffness. Although some patients' RCSPs are similar, their joint mechanical properties relevant to the reposition ability may be diverse (e.g., muscular spasticity and ligamentous flexibility) [34]. The present findings potentially implied that RCSPs might not be efficiently correlated with the mechanism of foot deformity in CP children and therefore not suitable as the primary principle for the development of treatment.

Although RCSP is a morphological index under a loading condition, ΔRCSP could indicate the morphological change under different loadings, which means that ΔRCSP could partially reflect the ankle stiffness in the coronal plane of the heel. The results indicated that STJ stiffness had significant negative correlations with ΔRCSP1w−0w (−0.397, *p* = 0.014) and ΔRCSP0.5w−0w (−0.365, *p* = 0.024). The correlation factors were small, because ΔRCSP reflected the calcaneal deformation in response to the vertical force on pelma, yet STJ stiffness reflected the calcaneal deformation in response to the reposition force.

The force-displacement curve was collected during the reposition process. The curve slope was small in the initial stage of loading; the small resistance in this stage was mainly contributed by the superficial skin and fat. With increasing displacement, the resistance rose linearly, which was contributed by the STJ structure and mechanical property. In the present study, STJ stiffness was defined as the slope of the line fitting the force-displacement curve, because the initial stage was short and had little influence on the calculated stiffness.

Although significant correlation was observed between STJ stiffness and CSI, only muscle tone and clonus had correlation with STJ stiffness. Previous study has reported that the muscle tone and muscle stiffness in CP children were higher than those in normal children [35]. Clonus is involuntary and rhythmic muscle contractions caused by a permanent lesion in upper motor neurons [36]. Therefore, both the muscle tone and clonus could increase the STJ constraint and contribute to the joint stiffness.

The present study has some limitations. First, in the process of measuring the STJ stiffness, the reposition force may influence the balance of the subject and influence the force-displacement data. However, the valgus deformities in this study were at mild and moderate level; the reposition forces were relatively small and had slight influence on the results. An improvement of the method is still necessary to decrease the potential deviation in our future study. Secondary, since the subjects were young children, the degree of their cooperation would also affect the measurements.

## 5. Conclusion

The present study quantified two indexes of the foot valgus in 38 feet of spastic CP children, the STJ stiffness and RCSPs (in three loading conditions). STJ stiffness had no correlation with RCSPs, yet it had negative correlation with the change of RCSP under different loading conditions (ΔRCSP_1w−0w_ and ΔRCSP_0.5w−0w_). STJ stiffness was also correlated with the composite spasticity index (CSI), implying that this index had an advantage in reflecting the mechanism of valgus deformity and should be considered as a necessary measurement of foot valgus in CP children. The present method for quantification of STJ stiffness could improve the accuracy in the diagnosis and classification of foot deformity and may help increase the understanding of the biomechanical factors in foot deformity rehabilitation.

## Figures and Tables

**Table 1:** CSIs of 19 children with 38 feet.

Foot number	Tendon jerk (0–4)	Muscle tone (0–8)	Clonus (1–4)	CSI (1–16)
1	2	4	1	**7**
2	3	6	2	**11**
3	2	4	1	**7**
4	3	6	1	**10**
5	2	4	1	**7**
6	2	2	1	**5**
7	2	4	1	**7**
8	3	6	2	**11**
9	3	6	1	**10**
10	3	6	1	**10**
11	2	4	1	**7**
12	3	6	1	**10**
13	2	2	1	**5**
14	3	6	1	**10**
15	2	2	1	**5**
16	2	4	1	**7**
17	2	4	1	**7**
18	2	4	1	**7**
19	2	2	1	**5**
20	2	2	1	**5**
21	3	6	1	**10**
22	1	2	1	**4**
23	2	4	1	**7**
24	3	8	2	**13**
25	3	6	1	**10**
26	2	4	1	**7**
27	3	8	2	**13**
28	3	6	1	**10**
29	2	2	1	**5**
30	3	6	1	**10**
31	1	2	1	**4**
32	2	4	1	**7**
33	3	8	2	**13**
34	2	4	1	**7**
35	3	8	2	**13**
36	3	6	1	**10**
37	2	2	1	**5**
38	3	6	1	**10**

**Table 2:** A Spearman correlation matrix of 8 factors.

	CSI	STJ stiffness	RCSP_1w_	RCSP_0.5w_	RCSP_0w_	ΔRCSP (1w − 0.5w)	ΔRCSP (1w − 0w)	ΔRCSP (0.5w − 0w)
CSI	∖	**r** = 0.343^*∗*^ (**p** = 0.035)	*r* = −0.048 (*p* = 0.773)	*r* = −0.010 (*p* = 0.953)	*r* = 0.135 (*p* = 0.419)	*r* = −0.049 (*p* = 0.771)	*r* = −0.241 (*p* = 0.144)	*r* = −0.190 (*p* = 0.254)
STJ stiffness	**r** = 0.343^*∗*^ (**p** = 0.035)	∖	*r* = −0.233 (*p* = 0.160)	*r* = −0.191 (*p* = 0.250)	*r* = 0.247 (*p* = 0.135)	*r* = −0.171 (*p* = 0.304)	**r** = −0.397^*∗*^ (**p** = 0.014)	**r** = −0.365^*∗*^ (**p** = 0.024)
RCSP_1w_	*r* = −0.048 (*p* = 0.773)	*r* = −0.233 (*p* = 0.160)	∖	**r** = 0.882^*∗∗*^ (**p** < 0.001)	*r* = 0.276 (*p* = 0.093)	*r* = 0.117 (*p* = 0.485)	**r** = 0.520^*∗∗*^ (**p** = 0.001)	**r** = 0.521^*∗∗*^ (**p** = 0.001)
RCSP_0.5w_	*r* = −0.010 (*p* = 0.953)	*r* = −0.191 (*p* = 0.250)	**r** = 0.882^*∗∗*^ (**p** < 0.001)	∖	*r* = 0.277 (*p* = 0.093)	*r* = −0.283 (*p* = 0.085)	**r** = 0.430^*∗∗*^ (**p** = 0.007)	**r** = 0.602^*∗∗*^ (**p** < 0.001)
RCSP_0w_	*r* = 0.135 (*p* = 0.419)	*r* = 0.247 (*p* = 0.135)	*r* = 0.276 (*p* = 0.093)	*r* = 0.277 (*p* = 0.093)	∖	*r* = −0.175 (*p* = 0.294)	**r** = −0.621^*∗∗*^ (**p** < 0.001)	**r** = −0.565^*∗∗*^ (**p** < 0.001)
ΔRCSP (1w − 0.5w)	*r* = −0.049 (*p* = 0.771)	*r* = −0.171 (*p* = 0.304)	*r* = 0.117 (*p* = 0.485)	*r* = −0.283 (*p* = 0.085)	*r* = −0.175 (*p* = 0.294)	∖	*r* = 0.299 (*p* = 0.068)	*r* = −0.081 (*p* = 0.628)
ΔRCSP (1w − 0w)	*r* = −0.241 (*p* = 0.144)	**r** = −0.397^*∗*^ (**p** = 0.014)	**r** = 0.520^*∗∗*^ (**p** = 0.001)	**r** = 0.430^*∗∗*^ (**p** = 0.007)	**r** = −0.621^*∗∗*^ (**p** < 0.001)	*r* = 0.299 (*p* = 0.068)	∖	**r** = 0.899^*∗∗*^ (**p** < 0.001)
ΔRCSP (0.5w − 0w)	*r* = −0.190 (*p* = 0.254)	**r** = −0.365^*∗*^ (**p** = 0.024)	**r** = 0.521^*∗∗*^ (**p** = 0.001)	**r** = 0.602^*∗∗*^ (**p** < 0.001)	**r** = −0.565^*∗∗*^ (**p** < 0.001)	*r* = −0.081 (*p* = 0.628)	**r** = 0.899^*∗∗*^ (**p** < 0.001)	∖

^*∗*^ *p* < 0.05; ^*∗∗*^*p* < 0.01.

**Table 3:** Correlations between CSI subindexes and the valgus indexes.

	STJ Stiffness	RCSP_1w_	RCSP_0.5w_	RCSP_0w_	ΔRCSP (1w − 0.5w)	ΔRCSP (1w − 0w)	ΔRCSP (0.5w − 0w)
Tendon jerk	*r* = 0.284 (*p* = 0.084)	*r* = −0.009 (*p* = 0.960)	*r* = −0.005 (*p* = 0.975)	*r* = 0.060 (*p* = 0.720)	*r* = 0.085 (*p* = 0.611)	*r* = −0.137 (*p* = 0.413)	*r* = −0.139 (*p* = 0.406)
Muscle tone	*r* = 0.335^*∗*^ (*p* = 0.040)	*r* = −0.034 (*p* = 0.839)	*r* = 0.026 (*p* = 0.878)	*r* = 0.121 (*p* = 0.469)	*r* = −0.090 (*p* = 0.590)	*r* = −0.215 (*p* = 0.194)	*r* = −0.146 (*p* = 0.381)
Clonus	*r* = 0.392^*∗*^ (*p* = 0.015)	*r* = 0.257 (*p* = 0.120)	*r* = 0.224 (*p* = 0.176)	*r* = 0.395^*∗*^ (*p* = 0.014)	*r* = −0.069 (*p* = 0.680)	*r* = −0.211 (*p* = 0.204)	*r* = −0.165 (*p* = 0.324)

^*∗*^ *p* < 0.05.
